# Living Space and Job Prospects and Their Relationship with Subjective Well-Being during COVID-19 Confinement in Spain: The Mediator Role of Resilience

**DOI:** 10.3390/ijerph18179198

**Published:** 2021-08-31

**Authors:** Fernando Molero, Patricia Recio, Encarnación Sarriá

**Affiliations:** 1 Faculty of Psychology, National University for Distance Education (UNED), 28040 Madrid, Spain; reciop@psi.uned.es (P.R.); esarria@psi.uned.es (E.S.); 2 Joint Research Institute IMIENS, 28029 Madrid, Spain

**Keywords:** COVID-19, confinement, living space, job prospects, subjective psychological well-being, resilience

## Abstract

The objective of this study was to examine the relationships of participants’ home characteristics (living space) and job prospects after the coronavirus disease 2019 (COVID-19) pandemic to their subjective psychological well-being (SWB) (in terms of both affective and cognitive aspects). We also examined the role of participants’ resilience as a possible mediator in the relationships among the aforementioned variables. The sample comprised 474 Spanish adults who completed an online questionnaire between 14 and 24 April 2020, when COVID-19 confinement was very strict in Spain. We proposed a path analysis model including the described variables. The model presented a good fit (χ^2^ = 7.41, df = 5, *p* = 0.376, comparative fit index = 0.996, Tucker–Lewis index = 0.987; root mean square error of approximation = 0.032). The results indicated that living space and future job prospects predicted resilience, which, in turn, was related to SWB. Moreover, the bootstrapping results revealed a mediating effect of resilience that showed indirect relationships between living space and SWB and between job prospects and SWB. Our results underline the importance of environmental (living space) and job-related variables to predict SWB as well as the mediating role that resilience may play during the confinement period.

## 1. Introduction

The coronavirus disease 2019 (COVID-19) pandemic has had many devastating effects. In terms of health, 209 million people have been infected with the virus, with more than 4.40 million deaths (data as of 20 August 2021) [1]. According to the World Bank [2], the global economy contracted by 4.1% in 2020, which constitutes the worst recession since World War II. Finally, the pandemic and confinement measures to stop the spread of COVID-19 have had negative effects on people’s mental health and psychological well-being [3]. As Tedros Adhanom, Director-General of the World Health Organization, stated, ‘There are reports from countries and in the scientific literature that COVID-19 illness is increasingly associated with mental and neurological manifestations, including delirium, as well as anxiety, sleep disorders, and depression. In addition, COVID-19 is likely to exacerbate pre-existing mental health, neurological and substance use disorders, while limiting access for those in need of services.’ [4] (p. 129).

This research was conducted between 14 and 24 April 2020, in Spain, where severe confinement of the population was ordered on 15 March, remaining in full effect until 4 May [5,6,7]. The study was therefore conducted during the harshest lockdown phase, with the end of this extreme situation unknown. Confinement prohibited people from leaving the house except to carry out work considered essential, such as that relating to health, security, and food. The confined population could only leave their homes to buy food or medicine. Schools were closed, and children could not go out to parks. Practising sports or running in the streets or parks was also forbidden.

A review of the literature shows that COVID-19 confinement negatively influenced several aspects of individuals’ well-being [8,9,10,11,12,13]. It is important to identify which specific aspects of confinement are damaging to subjective psychological well-being, and which psychosocial variables may help individuals cope with these negative effects. In this research, we focused on two aspects that seem especially important in a confinement period: the space that an individual has available in their home (living space) and their job prospects after the pandemic.

The objective of this research was to analyse some of the factors related to psychological well-being in an unexpected traumatic situation. Specifically, we examined the relationships of the characteristics of the home (e.g., size and number of people living there during confinement) and the participants’ job prospects after the pandemic to their subjective psychological well-being (SWB) in terms of both affective and cognitive aspects. We also examined the role of resilience as a possible mediator in these relationships. 

### 1.1. Subjective Psychological Well-Being (SWB)

The study of well-being has a long tradition within psychology, and there are different ways to approach it [14]. In this study, we adopted the hedonic perspective, which focuses on experiences of pleasure and displeasure and on judgements concerning the positive and negative aspects of life. This kind of well-being is also known as subjective well-being (SWB). According to Diener [15] (p. 590), SWB includes people’s beliefs and feelings about whether they are leading desirable and rewarding lives. Research has identified two main aspects of SWB: an affective component, reflecting an individual’s predominant affective state (positive or negative), and a cognitive component, reflecting an overall assessment called life satisfaction [16]. In terms of the affective component of SWB, research shows that positive affect (PA) and negative affect (NA) are clearly separable components of SWB [17]. 

Regarding SWB during the COVID-19 confinement period, studies have shown that people who suffered confinement, compared with those who did not suffer it, reported greater psychological distress, anxiety, depressive symptoms, irritability and emotional exhaustion [12,13]. On the other hand, Blasco-Belled et al. [10], in a study conducted on a sample of 541 Spanish participants, found that people with high PA and low NA adopted more adaptive strategies to face the situation caused by COVID-19. In our research, we considered SWB a criterion variable that may be affected by some environmental (living space) and psychological variables (job prospects) during the confinement period. Paredes et al. [18], using a sample of 720 participants, found that the relationship between the perceived threat from COVID-19 and well-being was mediated by resilience.

### 1.2. Resilience

Resilience refers to a dynamic process encompassing positive adaptation within the context of significant adversity [19]. According to Bonanno [20], resilience after a loss or potential trauma is more common than is often believed. For this reason, it is important to study resilience in order to achieve a comprehensive understanding of human responses to stress and trauma [21]. Various studies have shown that resilience is associated with psychological well-being and mental health in various types of groups, such as people with physical disabilities [22], mothers of children with autism spectrum disorder [23], seropositive gay men [24] and students [25]. A meta-analysis, including 31,071 participants from 33 studies, showed that PA and, to a lesser extent, life satisfaction are positively related to resilience, while NA is negatively related to it [26].

COVID-19 and the related confinement measures implemented constitute stressful events of the first order that had very negative consequences for mental health and psychological well-being. Thus, in this context, studying the role of resilience can help prevent the effects of the pandemic and confinement. Various experts recognise the role of resilience in preventing the negative psychological effects of the pandemic [27]. However, because of the immediacy of the event, very few empirical studies have been carried out (or published to date) on this period of time. Among them, we can mention the study of Tan et al. [28], who, with a sample of 1871 Chinese college students, found that resilience had a strong positive relationship with psychological well-being during the pandemic. Paredes et al. [18] showed that the relationship between the perceived threat of COVID-19 and psychological well-being is mediated by resilience. On the other hand, a study on a sample of 617 Norwegian participants found a moderator effect of resilience on the effects of stress (anxiety and depression) [29]. Prime et al. [30] presented a theoretical model of intervention in which family resilience lessens the negative effects of COVID-19 on the psychological adjustment of children.

### 1.3. Housing Conditions and Psychological Well-Being

Before the pandemic, many studies showed a positive association between household size and psychological well-being [31,32,33]. In fact, the number of rooms per person was used as an indicator of quality of life in the European Quality of Life Survey. 

Although not many studies have been conducted in this regard, one can assume that confinement caused by COVID-19 has made the relationship between housing characteristics and psychological well-being more important than in a ‘normal’ situation. Thus, in the UK, according to research published by the National Housing Foundation, 31% of adults experienced mental or physical problems due to the lack of space and the characteristics of the home during confinement [34]. Likewise, a research study conducted on a sample of more than 8000 Italian students showed a strong association between the size of the dwelling and the appearance of depressive symptoms during confinement [35]. This study showed that depressive symptoms and the quality of the home also negatively affected performance in work done at home during confinement. In particular, social isolation and living in a small apartment without a predesigned workspace led to a loss of productivity.

As far as we know, there has been no published research that has directly explored the association between living space and resilience within the context of an ongoing significant adversity. However, Schwartz et al. [36] found an association between socioeconomic status and resilience in people with chronic mental health conditions. This association was mediated by so-called reserve-building activities, which stimulated individuals intellectually, culturally, and physically. Living space is clearly related to socioeconomic status [32] and having enough space in the house to exercise or ensure privacy when using the Internet may be related to the ability to tolerate confinement.

### 1.4. Job Prospects after the Pandemic

According to the Spanish Sociological Research Centre survey published in May 2020 (which collected data for April), 87.4% of the population believed that the employment and economic consequences of the COVID-19 crisis would be very serious [37]. According to those surveyed, the first and second most important problems in Spain were perceived to be unemployment (41.2%) and economic troubles (38.9%), respectively. When the question was shifted to the personal level, participants declared that their biggest problems were associated with the economic crisis (39.4%), the coronavirus (31.4%), and unemployment (30%).

In the same line of enquiry, a study conducted with a representative sample of the Spanish population in April 2020 [8] showed that aspects related to job maintenance during and after the COVID-19 crisis were of utmost importance to the psychological well-being of the participants.

Apart from the studies on the effects of the COVID-19 crisis, many studies have shown a negative relationship between unemployment and psychological well-being [38]. Hoang and Knabe [39] pointed out that one of the most consistent findings in the life satisfaction literature is that unemployment is negatively related to life satisfaction. For this reason, we assumed that optimism about maintaining a job after the pandemic would be positively related to both well-being and resilience.

### 1.5. Current Research

**Figure 1:**
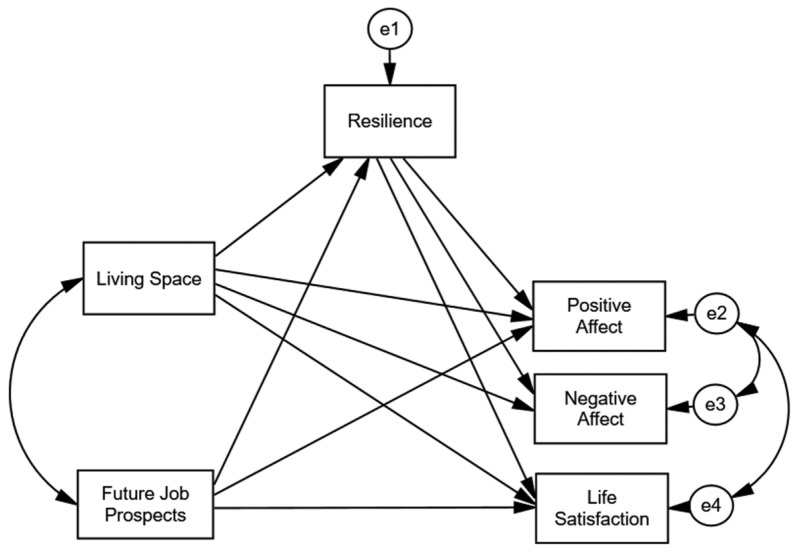
Hypothesised model.

In this research, we studied the relationships of two variables, a sociodemographic variable (living space) and a psychological variable (participants’ job prospects after the pandemic), to SWB. We also aimed to verify the mediating role of resilience in these relationships. Therefore, we formulated the model shown in Figure 1.

The model analysed, through structural equations, the relationships of housing conditions and future job prospects to SWB (positive and negative affect as well as life satisfaction) experienced by the participant, considering the mediating role that resilience could play in these relationships.

From the previous review, we formulated the following hypotheses:

**Hypothesis** **1.** *Living space is a predictor of subjective well-being, positively correlated to positive affect and life satisfaction and negatively correlated to negative affect.*

**Hypothesis** **2.** *Living space is a positive predictor of resilience.*

**Hypothesis** **3.** *Positive job prospects after the pandemic predict positive affect and life satisfaction (positively) and negative affect (negatively).*

**Hypothesis** **4.** *Positive job prospects after the pandemic positively predict resilience.*

**Hypothesis** **5.** *Resilience is a positive predictor of positive affect and life satisfaction and a negative predictor of negative affect.*

**Hypothesis** **6.** *Resilience mediates the relationship between predictors (living space or job prospects after the pandemic) and outcome variables (positive and negative affect or life satisfaction).*

## 2. Materials and Methods

### 2.1. Participants

The participants comprised 474 Spanish adults who completed an online questionnaire between 14 and 24 April 2020, when confinement was very strict in Spain and had been experienced by people for over a month. The sample was incidental. The percentage of missing values was below 3% (thus, imputation was not necessary), and the final sample size was 462 participants. 

### 2.2. Variables and Instruments

#### 2.2.1. Living Space

This index was obtained by dividing the self-reported size of the participant’s house by the number of residents during confinement.

#### 2.2.2. Job Prospects

Each participant answered an ad hoc question about her/his job prospects immediately after the pandemic, choosing from among five options: very bad (1), pretty bad (2), have not changed (3), good (4), and very good (5).

#### 2.2.3. Subjective Psychological Well-Being: Positive and Negative Affect

Measures of the affective component of SWB were obtained using the Spanish version of the Positive and Negative Affect Schedule (PANAS) [40,41], a 20-item scale with ten adjectives related to positive emotions (e.g., enthusiastic, attentive, proud) and another ten adjectives related to negative emotions (e.g., irritable, ashamed, scared). The participants provided responses to the items on a five-point Likert scale from 1 (‘not at all’) to 5 (‘extremely’) and were instructed to rate the emotions that they had felt in the last few weeks during confinement. The items were averaged together, resulting in a total score for each subscale. The total subscale scores ranged from one to five, with higher scores indicating higher levels of PA or NA. Cronbach’s alpha coefficient in our sample was 0.94 for the PA subscale and 0.90 for the NA subscale, indicating good levels of reliability.

#### 2.2.4. Subjective Psychological Well-Being: Life Satisfaction

To assess the cognitive component of SWB, the participants were asked to rate their degree of satisfaction with certain aspects (domains) of their lives based on domains of life satisfaction [42,43]. The following eight domains were considered: romantic relationship, family, finances, friendship, work, self, health, and life in general. The items were presented in the same order to all participants, who responded using a seven-point Likert-style scale (1 = totally dissatisfied, 4 = intermediately satisfied, 7 = very satisfied). We used the average of the items’ scores (ranging from one to seven) as a reflection of each participant’s satisfaction with his/her life. Cronbach’s alpha coefficient was 0.78 in our sample.

#### 2.2.5. Resilience

The Spanish version of the ten-item Connor-Davidson Resilience Scale (CD-RISC-10) [21,44], a self-administered questionnaire comprising ten of the original 25 items from the CD-RISC scale [45], was used to assess resilience. Example items are ‘I am able to adapt when changes occur’ and ‘Having to cope with stress can make me stronger’. The participants answered the items using a four-point Likert-style scale (from 1 = never to 4 = almost always). The items were averaged together, resulting in a CD-RISC total score (ranging from one to four). Higher scores represent a greater ability to bounce back from the challenges that can arise in life. Cronbach’s alpha coefficient in our sample was 0.86, which indicates that the scale had good internal consistency.

### 2.3. Procedure

Data were collected over a ten-day period between 14 and 24 April 2020, when there was mandatory confinement in Spain and people had already been isolated at home for over a month. Answers to the study were collected through an online questionnaire designed and hosted at www.qualtrics.com (accessed on 10 April 2020). We used convenience sampling with the snowball sampling technique. Students from the National University of Distance Education (UNED) were invited to participate in the investigation without any incentive being offered. They received an email sent by one of the researchers, a professor at their university, inviting them to participate voluntarily in research conducted by the Social Psychology department on ‘the personal experience during the situation of confinement by COVID-19’. The email contained a link to access the questionnaire. They were also invited to distribute the questionnaire link among friends, relatives, or acquaintances to recruit more participants. The participants were informed about the objectives and given guarantees of anonymity and confidentiality before providing their consent. 

### 2.4. Data Analysis

The descriptive characteristics were analysed using SPSS Statistics 24.0 (IBM, Armonk, NY, USA) [46]. The main analysis of this study consisted of testing a path analysis using AMOS version 24.0 (IBM, Armonk, NY, USA) [47], in which this model was hypothesised to illustrate the plausible mediating role of resilience in the relationship of living space and future job prospects to PA and NA as well as life satisfaction. The bootstrap method was applied with 10,000 repetitions, establishing a confidence interval of 95%. Bootstrapping is one of the most widely used methods to estimate mediation because it leads to more robust estimates than other mediation methods (such as the Sobel test) insofar as it is not affected by a lack of normality in the residual distribution [48]. 

The aforementioned path analyses were estimated via the maximum likelihood method. To evaluate the overall fit of the model to the data, several indices proposed by Hu and Bentler [49] and Kline [50] were calculated in this study: the chi-squared statistic (χ^2^), the comparative fit index (CFI), the Tucker-Lewis index (TLI), and the root mean square error of approximation (RMSEA). For the CFI and TLI, values over 0.90 indicate an acceptable fit, whereas values over 0.95 indicate a good fit. RMSEA values near 0.05 indicate an excellent fit, whereas values between 0.05 and 0.08 indicate an acceptable fit [49,50].

## 3. Results

### 3.1. Preliminary Analyses

Participants’ ages ranged between 18 and 72 years (M = 36.1; SD = 12.4), and our sample contained a majority of women (73.4% women and 22.3% men). A large percentage of the sample reported that a relative or close friend had been ill with COVID-19 (45.7%). Other information on the participants’ housing conditions during mandatory confinement and working conditions is shown in Table 1. As already indicated, students from the UNED were invited to participate in the investigation. Two specific characteristics of the UNED should be highlighted to better understand the characteristics of the sample. These are the national scope of this university and its teaching-learning system (blended learning) that is compatible with work activity. This explains the participants’ mean age and the high percentage who reported being employed.

The descriptive statistics and bivariate correlations among all the observed variables are presented in Table 2. The participants’ mean scores for resilience (t (461) = 22.16, *p* < 0.001) and life satisfaction (t (461) = 22.67, *p* < 0.001) were both above the midpoint of the scale, whereas for PA, the mean scores were at approximately the midpoint of the scale (t (461) = −0.16, *p* = 0.866). In addition, their mean scores for NA (t (461) = −20.05, *p* < 0.001) and future job prospects (t (461) = −7.78, *p* < 0.001) were below the midpoint of the scale.

Living space was positively correlated with resilience (*p* < 0.001) and future job prospects (*p* = 0.001) and negatively correlated with NA (*p* = 0.002). Future job prospects were positively correlated with resilience (*p* < 0.001), PA (*p* < 0.001), and life satisfaction (*p* < 0.001) and negatively correlated with NA (*p* < 0.001). Finally, resilience was positively correlated with PA (*p* < 0.001) and life satisfaction (*p* < 0.001) and negatively correlated with NA (*p* < 0.001). 

### 3.2. Model Testing

**Figure 2:**
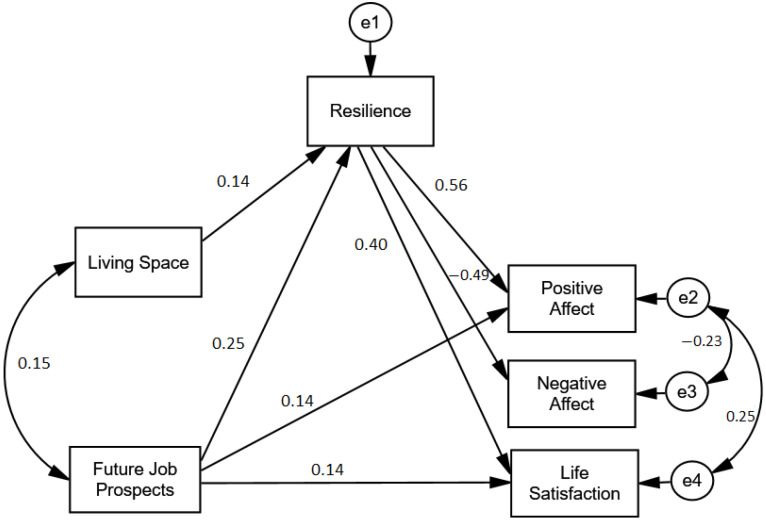
Standardised path coefficients among the variables. All the path coefficients are statistically significant.

The hypothetical model was tested among the sample and demonstrated an acceptable fit to our data (χ^2^ = 2.41, df = 4, *p* = 0.12, CFI = 0.997, TLI = 0.962, RMSEA = 0.055). However, none of the three direct effects between living space and SWB were significant. This is consistent with the assumption of complete mediation, and therefore we excluded these three insignificant direct effects. The same applied for the direct effect between future job prospects and NA, which was also removed. The final model (see Figure 2) improved the fit (χ^2^ = 7.41, df = 5, *p* = 0.376, CFI = 0.996, TLI = 0.987, RMSEA = 0.032).

The results indicated that living space predicted resilience (β = 0.14, *p* = 0.002), which, in turn, was positively related to life satisfaction (β = 0.40, *p* < 0.001) and PA (β = 0.56, *p* < 0.001) and negatively related to NA (β = −0.49, *p* < 0.001). Moreover, we found a significant and positive association between future job prospects and resilience (β = 0.25, *p* < 0.001), PA (β = 0.14, *p* < 0.001), and life satisfaction (β = 0.14, *p* < 0.001). A summary of the direct and indirect effects is provided in Table 2; resilience played a mediating role in all relationships.

We observed an indirect relationship between living space and PA, mediated by resilience. To analyse this, we restricted the paths from living space to resilience and from resilience to PA to 0 in the direct model. In this case, the direct relationship between living space and PA was β = 0.113 (*p* < 0.001), and dropped to a non-significant value of β = −0.013 (*p* = 0.482) when resilience was introduced into the model. The bootstrapping results revealed that the mediating effect of resilience produced an indirect relationship (β = 0.080, *p* < 0.001; 95% CI: 0.027, 0.133). The same applied for the mediating role of resilience in the relationships between living space and NA and between living space and life satisfaction (see Table 3).

We also found an indirect relationship between job prospects and PA that was mediated by resilience. In this case, the direct relationship between job prospects and PA (β = 0.309, *p* < 0.001) decreased when resilience was introduced as a mediator, but remained a significant value (β = 0.154, *p* < 0.001). The bootstrapping results revealed that the mediating effect of resilience gave rise to significant indirect relationships (β = 0.139, *p* < 0.001; 95% CI: 0.088, 0.189). The same applied for the mediating role of resilience in the relationships between future job prospects and NA and between future job prospects and life satisfaction (see Table 3).

## 4. Discussion

The main objective of this study was to analyse the association between two environmental and psychosocial variables (living space and job prospects after the pandemic) and the participants’ SWB during the most difficult period of confinement in Spain.

Regarding participants’ SWB, results revealed that, on average, the levels of positive and negative affect among the sample were good, with positive affect scores in the midpoint of the scale and negative affect scores below the midpoint of the scale. These results are similar to those obtained by Beato et al. [9] in their study on the Portuguese population during lockdown. Portugal and Spain are very close both geographically and culturally. Furthermore, the population of both countries suffered strict confinement at the same time. Our sample also showed good levels of resilience and life satisfaction, even though participants had low levels of optimism about keeping their job after the pandemic crisis.

Many researchers have focused on the significant influence of living space on several aspects of SWB [31,32,33]. Moreover, they found that when people were prohibited from leaving the house during the confinement period, the characteristics of their homes increased in relevance. For example, Amerio et al. [35], in a study with a sample of more than 8000 Italian students, found a strong association between the size of the dwelling and the appearance of depressive symptoms during confinement. Regarding Hypothesis 1, the results showed that the individuals’ living spaces predicted positive and negative affect and life satisfaction, although in the model testing none of the three direct effects between living space and SWB were significant when the mediator was included. This was consistent with the assumption of complete mediation through resilience. Several aspects may be implied in the beneficial consequences of living space on well-being. For example, having more space and thus having more privacy to carry out different activities (e.g., talking on the phone or online, listening to music, reading a book, performing physical exercise) may help to enhance positive emotions and decrease negative ones. Moreover, having a larger home is related to a higher level of income or education, variables that were also positively related to SWB. 

In Hypothesis 2, we predicted a positive association between living space and resilience. Resilience consists of a dynamic process encompassing positive adaptation within the context of significant adversity. Although no research study has directly explored the association between living space and resilience, some studies have found that socioeconomic status, a variable closely associated with living space, may be related to better adaptation to a traumatic situation [32,36]. 

Regardless of the COVID-19 crisis, many studies have shown the importance of employment situation on psychological well-being [38]. Hoang and Knabe [39] pointed out that one of the most consistent findings in the life satisfaction literature is that unemployment is negatively related to life satisfaction. During confinement, most people were worried about their job prospects or job expectations after the pandemic. A Spanish representative survey [8] found that job situation was one of the most important variables related to psychological distress. Another study, conducted with a sample of 2008 Spanish participants, showed that post-confinement work expectancy and pre-confinement working conditions were important variables related to well-being in confined populations [51].

For this reason, we assumed that optimism about maintaining a job after the pandemic was positively related to well-being and constituted a factor in resilience [26]. Our results show that future job prospects were a positive predictor of positive affect and life satisfaction (Hypothesis 3) and predicted resilience as well (Hypothesis 4). 

A review of literature on the effects of the pandemic shows the positive effects of resilience on mental health and well-being [18,27,29,52]. Our results point in this direction, showing not only a highly positive association between resilience and both positive affect and life satisfaction and an inverse relationship between resilience and negative affect (Hypothesis 5), but also the mediator role of resilience.

Regarding the hypothesis on the mediator role of resilience (Hypothesis 6), the tested model presented a good fit. The model parameters show that environmental comfort during confinement (living space) and job prospects were positively correlated with resilience, which, in turn, had a significant direct relationship with positive affect and life satisfaction as well as an inverse relationship with negative affect. The results of the mediational analysis report complete mediation through resilience in the relationship between living space and SWB. However, the relationship between job prospects and SWB was only partially mediated by resilience. These mediations may be explained by the strength of the correlations among the variables. The association between living space and SWB was not very strong, and the inclusion of resilience in the equation made this relationship non-significant (total mediation). By contrast, the association between job prospects and SWB was stronger, and resilience caused it to decrease but remain significant (partial moderation). Our results are compatible with those obtained by Zager et al. [53] that found that resilience mediated the relationships between the Big Five and both SWB and perceived stress. 

This study has several limitations. The cross-sectional nature of the research needs to be considered. Although path analysis is an advanced statistical approach, causal inferences about the relationships among the studied constructs cannot be made. Regarding the generalisability of the results, although the sample size is large enough and its composition is heterogeneous, the sample was not representative of all of the general population because of its non-probabilistic character. Another relevant limitation lies in the model itself and its explanatory capacity. The variables included in the model are only a small part of the large set of variables that could influence well-being during the confinement period. Special mention should be made of certain sociodemographic variables such as socioeconomic status, educational level, and professional profile, which are closely related to the predictors considered in this study. However, other conditions such as the possibilities of social relations during confinement—that is to say, living with a partner, family or alone or the frequency of social contact, even through telematic means—are variables that could intervene in the psychological effects of confinement and were not analysed in this study.

Despite these limitations, this study may help contribute to the literature as it goes beyond verifying the psychological impact of the pandemic and instead seeks to understand the mechanisms and processes involved. 

The findings have several implications. Firstly, they underline the importance of a comfortable physical environment for psychological well-being. Indeed, having enough living space in the house and a safe and healthy neighbourhood is always important, but in a confinement situation these aspects are particularly relevant. Certain possibilities of relief (such as authorising walks or the use of parking lots by children) in the measures restricting mobility to contain the pandemic may be of great importance for families with worse living space conditions. Living space is clearly related to socioeconomic status [27]. Thus, the consequences of restrictive measures such as confinement could be worse for families with fewer economic resources, which in turn may also be more affected by threats to their employment opportunities in the face of a possible economic crisis resulting from the pandemic. Our results also show that positive job prospects are important. Therefore, people working in areas that have been less harmed by the pandemic, or with better prospects after it, have a greater probability of conserving an adequate level of SWB. This may lead to the conclusion that government employment protection measures are not only relevant for the future after the pandemic but also for the psychological adaptation of the population during the containment periods of the pandemic. Finally, there is an individual psychological factor, resilience, that contributes to the maintenance of SBW in traumatic situations. It is important to continue studying both the individual antecedents of resilience [54] and the possibility of training this ability before or during the time in which the traumatic event is taking place [55].

## 5. Conclusions

The COVID-19 pandemic has been a traumatic event for millions of people all over the world. Both the pandemic itself and the confinement measures to stop the spread of COVID-19 have had significant negative effects on people’s mental health and psychological well-being. In this context, it is important to explore which factors may contribute to maintaining an adequate level of SWB during the pandemic. Our research demonstrates that SWB was related to environmental factors (living space) and to job expectations after the pandemic, with personal resilience playing a mediating role. 

Overall, our findings highlight the relevance of resilience as a psychological factor that mediated the relationships between environmental conditions (living space) or the subjective perceptions of one’s own employment perspectives after the pandemic and psychological well-being. These findings allow us to identify factors that deserve attention to the extent that their strengthening would promote better psychological adaptation during the pandemic. 

The unexpected COVID-19 pandemic has had very detrimental effects on many levels; however, it has given us the opportunity to study aspects of human behaviour under traumatic conditions. We expect that the generated knowledge may be useful for future traumatic events as well.

## Figures and Tables

**Table 1:** Sociodemographic characteristics.

Characteristic	% (*n*)
**Number of residents in home during confinement**	
1 individual	7.4 (34)
2 individuals	37.2 (172)
3 individuals	24.2 (112)
4 individuals	22.1 (102)
5 or more individuals	3.4 (39)
**Housing type**	
Interior flat	5.6 (26)
Exterior flat without balcony or terrace	20.1 (93)
Exterior with balcony	22.5 (104)
Exterior with terrace	28.1 (130)
Chalet with garden	23.6 (109)
**Employment prior to confinement**	
Self-employed	7.1 (33)
Employed	61.5 (284)
Unemployed	7.8 (36)
Student	20.1 (93)
Retired	3.5 (16)
**Changes in employment status because of confinement**	
No, I am in essential services	19.9 (91)
No, I am a student, unemployed or retired	31.2 (143)
Yes, I am now working remotely	23.4 (107)
Yes, I am in a temporary or definitive suspension of contract	25.6 (117)

**Table 2:** Means (M), standard deviations (SD), and Pearson correlation coefficients among the variables in the study (*n* = 462).

	Scores Range	M	SD	2	3	4	5	6
1. Living Space	7.5–280	43.04	27.97	0.15 *	0.18 *	0.11	−0.14 *	0.10
2. Future Job Prospects	1–5	2.67	0.90		0.27 *	0.30 *	−0.20 *	0.25 *
3. Resilience	1–4	3.02	0.50			0.59 *	−0.49 *	0.44 *
4. Positive Affect	1–5	2.99	0.93				−0.47 *	0.46 *
5. Negative Affect	1–5	2.24	0.81					−0.28 *
6. Life Satisfaction	1–7	5.09	1.03					

* *p* < 0.003 (Bonferroni correction).

**Table 3:** Results of mediational analysis.

Mediational Analysis	Direct Beta without Mediator	Direct Beta with Mediator	Indirect Beta [CI]
LS → R → PA	0.113 *	−0.013	0.080 ** [0.027–0.133]
LS → R → NA	−0.142 **	−0.055	−0.070 ** [−0.121–0.024]
LS → R → LifeS	0.100 *	0.003	0.057 ** [0.020–0.098]
FJP → R → PA	0.309 **	0.154 **	0.139 ** [0.088–0.189]
FJP → R → NA	−0.203 **	−0.070	−0.122 ** [−0.170–0.075]
FJP → R → LifeS	0.251 **	0.143 **	0.099 ** [0.060–0.146]

Notes: LS = living space; R = resilience; PA = positive affect; NA = negative affect; FJP = future job prospects; LifeS = life satisfaction. * *p* < 0.05; ** *p* < 0.01.

## Data Availability

The data presented in this study are available on request from the corresponding author.
